# Clinical validation of the Standardized Patient Evaluation of Eye Dryness Questionnaire in European Portuguese in a non-clinical sample

**DOI:** 10.1007/s10792-025-03437-1

**Published:** 2025-02-07

**Authors:** Eva Ramalho, Ivo Soares, Francisco Miguel Brardo, Amélia Fernandes Nunes

**Affiliations:** 1 University of Beira Interior, Covilhã, Portugal; 2 Center for Research in Health Sciences (CICS), University of Beira Interior, Covilhã, Portugal; 3 Clinical and Experimental Center in Vision Sciences (CCECV), University of Beira Interior, Covilhã, Portugal

**Keywords:** Dry eye disease, DED, SPEED questionnaire, Surveys and questionnaires, OSDI questionnaire

## Abstract

**Introduction:**

The aim of this study is to determine the best cut-off value for the Standardized Patient Evaluation of Eye Dryness Portuguese version questionnaire and verify possible differences between the questionnaire score and different age groups.

**Methods:**

The clinical validation of the Standardized Patient Evaluation of Eye Dryness Portuguese version was performed by completing this and the Ocular Surface Disease Index in Portuguese version questionnaire, in 180 volunteers (68.9% female), with an age range of 19–96 years.

**Results:**

An excellent degree of reliability was found between the Standardized Patient Evaluation of Eye Dryness Portuguese version and the Ocular Surface Disease Index Portuguese version questionnaires (Cronbach’s Alpha = 0.824). The Receiver Operating Characteristic curve analysis revealed a cut-off of 8 points (Sensitivity of 71.2% and Specificity of 71%). Furthermore, the area under the curve of the Receiver Operating Characteristic curve was 0.813, indicating that Standardized Patient Evaluation of Eye Dryness Portuguese version questionnaire is a good diagnostic tool and interchangeable with the Ocular Surface Disease Index questionnaire.

**Conclusion:**

The Standardized Patient Evaluation of Eye Dryness Portuguese version questionnaire is a reliable tool for the detection of dry eye symptoms in the Portuguese population with a broad age range.

## Introduction

Dry eye disease (DED) is one of the most common ocular morbidities and a public health problem, affecting ocular comfort and visual performance. The literature lists several risk factors for DED, including aging, gender, ocular and systemic diseases, and the use of systemic drugs. Furthermore, modern-day life, with its extensive use of digital screens, has become a prominent source of complaints related to DED [1]. The prevalence of risk factors for developing dry eye symptoms is becoming increasingly evident among young populations. This exposure to risk factors can harm their quality of life, functional abilities, and productivity [2–4].

There is strong evidence that questionnaires based on the intensity and frequency of symptoms of dry eye are useful tools in helping to diagnose DED [5]. On the other hand, the literature also reports a low correlation between clinical signs and symptoms of DED [3, 6]. The Ocular Surface Disease Index (OSDI) and the Standard Patient Evaluation of Eye Dryness (SPEED) questionnaires are some of the most widely used in clinical practice and research [7]. Although the OSDI questionnaire is considered the gold-standard questionnaire [6], both questionnaires were evaluated in clinical and non-clinical studies [3, 8–10] and considered similar and interchangeable in clinical practice [8]. Additionally, both surveys have been adapted and translated for use in a variety of languages and cultural contexts [7, 11, 12].

The effectiveness of DED detection by the questionnaires depends on the correct choice of a specific cut-off point or scoring value [3, 8]. For the SPEED questionnaire, the literature agrees that the best scoring value is between 4 and 6, [3, 8, 13] although it is also possible to find an alternative value of 11 [14]. This higher score was found in a study with several age groups and ethnic backgrounds [14]. The cut-off point was studied by comparing subjective symptoms from 5 questionnaires and objective clinical signs (NIBUT, tear osmolarity, and ocular surface coloration), [14] while the studies that obtained cut-off points 4 and 6 only used the OSDI questionnaire [3, 8, 13].

For the OSDI questionnaire, the literature indicates that there is good consistency with the same scoring value (13 points) for all age groups, with some authors hypothesizing that it could be higher in a young population [9]. Weng et al. present possible reasons for this, such as the higher corneal nerve density in younger people, with a lower pain threshold, while in older people the corneal nerve density tends to decrease [9]. It should be noted that this hypothesis lacks scientific solidity, as there are few studies on DED in childhood or adolescence. Of the 28 clinical studies that employed the SPEED questionnaire, only 2 were conducted in a young population [15].

This research aims to determine the most appropriate scoring value for a non-clinical population with the Portuguese version of the SPEED questionnaire. Additionally, it was also studied differences in symptom scores between genders and age groups.

## Material and methods

### Population

This is an observational and cross-sectional study, with a non-probability sample and with recruitment by convenience. The study population includes students and staff from the University of Beira Interior, users of a Senior University, and daycare users of a social support institution. The questionnaires were self-administered at the Clinical and Experimental Center in Vision Sciences (CCECV) and at the daycare social institution. All participants gave their informed consent to participate in the study. All procedures were conducted in accordance to the Declaration of Helsinki. This study was approved by the University of Beira Interior Ethics committee. The data for this study were collected using two questionnaires: the Portuguese versions of the OSDI and SPEED questionnaires [11, 12]. In addition, sociodemographic data, including age, gender, education level, and professional activity, were collected to characterize the sample. The study was conducted between January 2023 and April 2023. The study considered Portuguese citizens over 18 years old with the necessary cognitive abilities to complete the questionnaires. All questionnaires were completed on paper forms under the researcher’s supervision. A total of 180 subjects aged 19–96 years were considered. For the gender group, 68.9% were female; for the age group, 52.2% were under 50 years old; and for the occupation group, 44.4% were students, 26.7% were working adults, and 28.9% were retired.

### Questionnaires description

The OSDI questionnaire, developed by Allergan (Irvine, California, EUA) in 1997, is widely recognized as the gold standard in clinical trials [8]. It consists of 12 questions divided into three sections: symptom occurrence, impact on vision-related quality of life, and influence of environmental factors [3, 15]. The scoring system, which has a range of 0 to 100 points, is computed by dividing the total points plus 25 by the total number of questions answered [16]. In both clinical and non-clinical populations, a score of ≥ 13 denotes symptomatic individuals and a score of < 13 denotes non-symptomatic individuals [3, 8, 15, 17].

The SPEED questionnaire was developed by Tear-Science (Morrisville, North Carolina, EUA) in 2005 and is used in clinical and non-clinical studies [7]. The short form of the SPEED questionnaire consists of 8 questions that assess the frequency (4 questions) and severity (4 questions) of symptoms. The final score ranges from 0 to 28 points, and higher scores represent more severe symptoms. The full form, which is primarily used in clinical practice, also evaluates the timing of symptom onset over a 3-month period [3, 8]. The literature indicates cut-off points such as 4 or 6 for the SPEED questionnaire [3, 8, 13]. A cut-off point of 11 is also mentioned in a study comparing several questionnaires [14].

The Brazilian Portuguese version of the OSDI questionnaire,[11], was used as an initial basis for a pre-test and subsequent linguistic adaptation. All items of the Brazilian Portuguese pre-test of the OSDI questionnaire were easily understandable, indicating that no linguistic adjustments or adaptations were required. The short version of the SPEED-vP questionnaire[12], was used to determine the optimal cut-off point.

### Data analysis

To assess gender and age as potential risk factors, the sample was divided into two gender groups (male and female) and two age groups (< 50 years and ≥ 50 years). The separation of the sample into two groups allowed us to perform an inter-gender and inter-age analysis to determine possible SPEED-vP score differences. The literature suggests a gradual increase in symptoms and clinical signs of DED after the age of 50, potentially associated with hormonal changes [18, 19]. Furthermore, the sample was stratified into 3 groups based on occupation, namely students, working adults, and retired people. This stratification aimed to investigate differences in questionnaire scores between these groups, considering that their habits and lifestyles may differ.

### Statistical treatment

The statistical treatment of the data was carried out with IBM® SPSS Statistics version 28 statistical software. Descriptive statistics, means, standard deviation, and frequencies were used to characterize the sample and to study the variables under analysis. The Cronbach’s Alpha coefficient was used to evaluate the internal reliability of the SPEED-vP and OSDI questionnaires. Since the distribution of variables normality (using the Shapiro–Wilk test) was not obtained, statistical inference was made using non-parametric tests. The appropriate cut-off value for the SPEED-vP questionnaire was determined by analyzing the ROC curve. The point in the ROC curve where sensitivity and specificity reach their best values was defined as the cut-off value [20].

## Results

### Study of symptomatic differences

The mean SPEED-vP score was 8.2 ± 4.8 for the total sample. Table 1 shows the results stratified by gender, age, and occupation. The Cronbach Alpha coefficient analyses revealed 0.824 and 0.835 for SPEED-vP and OSDI, respectively. These results show that the OSDI and SPEED-vP questionnaires have good internal reliability. Differences in SPEED-vP scores according to gender, age group, and occupation were studied using the Kruskal–Wallis test (more than two categories) or the Mann–Whitney test (two categories). Table 1 shows that no significant differences were found between the age, gender and occupation groups (*p* > 0.05).

**Table 1:** Demographic data of the volunteers

	Total sample	Age		Gender		Occupation		
		< 50 years	≥ 50 years	Male	Female	Students	Working adults	Retired
	180	94	86	56	124	80	48	52
Age (mean ± SD)	44.7 ± 23.5	24.1 ± 6.8	67.2 ± 11.7	41.5 ± 2.2	46.1 ± 24.1	21.8 ± 1.6	50.3 ± 10.1	74.7 ± 8.5
SPEED (mean ± SD)	8.2 ± 4.8	7.8 ± 4.2	8.6 ± 5.4	7.9 ± 4.9	8.3 ± 4.8	8.1 ± 4.1	7.7 ± 5.2	8.8 ± 5.5
Differences study (*p*-value)	–	0.378*		0.392*		0.465**		

SPEED, Standardized Patient Evaluation of Eye Dryness; SD, standard deviation

*Mann–Whitney test; **Kruskal–Wallis test

#### Study of the cut-off point

**Fig. 1:**
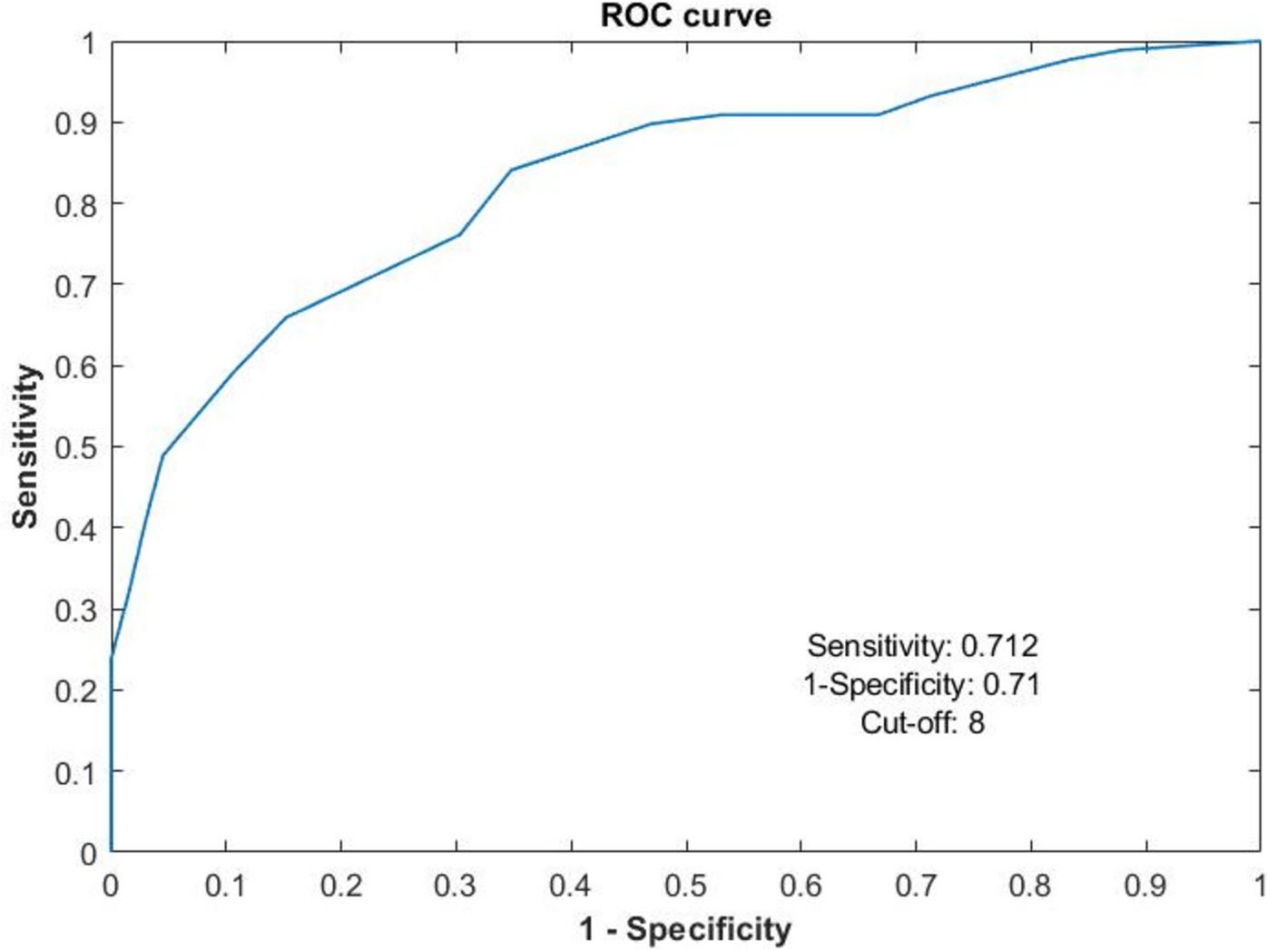
Operating characteristic curve of the receiver of the SPEED-vP questionnaire. (The ordinates of the graph show the sensitivity and the abscissa shows the 1-specificity; the indicated cut-off point is 8 with a sensitivity of 0.712 and specificity of 0.71, respectively)

To establish an appropriate cut-off value for the SPEED-vP questionnaire, participants were categorized according to the OSDI score as symptomatic (score ≥ 13) or asymptomatic (score < 13). Subsequently, the generated ROC curve using the results of the SPEED-vP questionnaire was created using the SPSS Statistics v28 (see Fig. 1). The analysis yielded a ROC of 0.813 (95% CI: 0.752–0.875). This result suggests that the SPEED-vP is a reliable test for dry eye symptom identification and can effectively diagnose DED as well as the OSDI questionnaire.

Based on Table 2, the SPEED-vP cut-off points that show adequate sensitivity and specificity are 7, 8, and 9. Since a cut-off point of 8 provides the best balance between sensitivity and specificity, this value was selected as the cut-off point for the SPEED-vP questionnaire.

**Table 2:** Possible cut-off values for SPEED-vP with acceptable sensitivity and specificity for the total sample and stratified by age groups

Score SPEED-vP	Total sample	
	Sensitivity (%)	Specificity (%)
4	91.9	33.3
5	90.1	46.4
6	87.4	52.2
7	79.3	65.2
**8**	**71.2**	**71**
9	62.2	85.5
10	55	89.9
11	46.8	95.7

Bold numbers represent the sensivity and specificity for the cut-off point

## Discussion

The objective of this study was to determine the best cut-off point to distinguish between asymptomatic and symptomatic subjects with dry eye using the SPEED-vP questionnaire. In our study sample, we identified a cut-off point of 8 on the SPEED-vP questionnaire as the most suitable (sensitivity of 71.2% and specificity of 71%). The mean SPEED-vP score obtained was 8.21 ± 4.8 and no statistically significant differences in SPEED-vP scores were observed between age, gender, or occupation groups.

Regarding the SPEED-vP questionnaire cut-off, there is some heterogeneity in the literature. Many studies report cut-off scores ranging from 4 to 6, with acceptable levels of sensitivity and specificity for classifying symptomatic dry eye [3, 8, 13]. Asiedu et al., in the Republic of Ghana considered a sample of over 600 subjects from a non-clinical population (university students) and found a cut-off point of 4 for the SPEED questionnaire (sensitivity of 74.2% and specificity of 76.7%) to distinguish symptomatic from asymptomatic individuals [8]. Similarly, Hashmani et al., in Pakistan found that a cut-off of 4 yielded a sensitivity of 73.4% and a specificity of 70.54% [3]. In another study, Asiedu et al. considered a sample of 650 university students and found a cut-off point of 6 [13]. Wang et al., in New Zeland, considered 211 non-clinical subjects and found a cut-off point of 11, with a sensitivity of 60% and specificity of 52% in DED diagnosis [14]. Geographic location, sample characteristics related to habits and lifestyles, and sample size may be factors contributing to these differences [3, 8, 13, 14]. One possible explanation for the discrepancies between studies is that Wang et al. [14], studied the SPEED cut-off point using objective clinical signs, while Asiedu et al. [8] and Hashmani et al. [3] used the OSDI (symptoms) to determine the cut-off point.

In this work, the mean SPEED-vP score (8.21 ± 4.80) is higher than the reported mean scores in other studies conducted with the same tool and in non-clinical populations. Studies conducted with the SPEED questionnaire in its original language (English) have reported mean score values of 4.5 ± 4.7 [8]; 6.02 ± 4.6 [3] and 4.3 ± 4.8 [21]. However, when the questionnaire was adapted to new languages, different mean scores were observed. For example, Facchin et al. conducted in Italy, with an adapted version in Italian, reported a mean score of 7.68 ± 4.22 [22]. These findings suggest that SPEED questionnaires translated into different languages and cultures tend to have higher mean scores than the original SPEED questionnaire.

Similar results were observed with the OSDI questionnaire in its original and translated versions. The mean scores of the OSDI questionnaire in its original version (English) ranged from 10.6 to 22.4 [3, 8, 21]. When considering the adapted versions of the OSDI questionnaire, Miyuki et al. with the Brazilian version showed a mean score of 38.98 ± 28.10 [23], Garza-León et al. with the Spanish version had a mean score of 26.85 ± 20.79 [24], Bakkar et al. with the Arabic version of the OSDI presented a mean score of 32 ± 21.56 [25], and Midorikawa-Inomata et al. with the Japanese version had a mean score of 37.7 ± 22.2 [26]. It is worth noting that only the study by Lu et al. reported a mean score of 22.7 ± 13 for the Chinese version of the OSDI, which closely resembles the mean score of the original OSDI [27]. These results show that the mean scores of the OSDI questionnaire tend to differ when translated into different languages, frequently exceeding the mean score of the original version. However, further investigation is necessary because there are limited studies conducted with language-adapted versions of the SPEED questionnaire. In addition, the habits and lifestyles of the populations in each of these studies are different and can influence the results. When translating DED questionnaires into other languages, it is essential to carefully consider cultural nuances and differences and carry out extensive validation to guarantee accurate and trustworthy interpretations of the questionnaire results.

No statistically significant differences were found in the mean SPEED-vP scores between the groups stratified by age, gender, or occupation. Identical findings were reported by Sanches et al. regarding gender (*p* = 0.363) and age (*p* = 0.733), using the Portuguese version of the SPEED questionnaire [12]. Similar results were also observed by Xie et al. who found non-significant differences in age (*p* = 0.134) and gender (*p* = 0.706) [28]. Blackie et al. also reported non-significant differences in age (*p* = 0.7) [10]. These consistent results from various studies suggest that the mean SPEED-vP score is not significantly impacted by age, gender, or occupation.

In general, dry-eye symptoms exhibit minimal changes before age 50, a gradual increase after that age, and a greater increase after age 80 [18]. It is possible that significant differences in the mean questionnaire scores between age groups (< 50 years and ≥ 50 years) may not be easily observed, given the gradual nature of symptom changes after age 50. This implies that differences in symptom severity with age may be less pronounced and that in order to identify meaningful differences in the mean questionnaire scores, a larger sample size or more focused age groups may be required.

According to the findings of Stapleton et al., there is a slight gender difference in the prevalence of DED symptoms, although these become more noticeable with age [18]. The lack of significant differences between genders and the mean SPEED-vP score may explain our results. Considering the occupation item, research indicates that students tend to have a higher prevalence of DED symptoms [24, 29]. This is supported by the intense exposure to digital devices and the fact that screen time is a risk factor for DED by reducing blinking [30–32]. The working adult group in our study included university staff members who use digital devices daily. In addition, most subjects in the retired group are enrolled in multiple digital device activities at a senior university. As a result, digital habits between our groups may not vary significantly, leading to similar SPEED-vP symptomatic scores.

### Strengths and limitations

This study has several strengths. First, it was found that the SPEED-vP questionnaire exhibited excellent internal consistency and performed well as a diagnostic tool, comparable to the OSDI questionnaire. It should be noted that the SPEED-vP questionnaire is the only dry eye diagnostic tool adapted to European Portuguese, scientifically validated. In addition, this study established a cut-off point of 8 for the SPEED-vP questionnaire in the Portuguese population, using the OSDI as a reference.

This study is not without limitations. First, the considered sample was non-probability and recruited through convenience, which limits results generalization. Moreover, the distribution of participants across gender and occupation groups was uneven, potentially introducing bias. It is worth noting that the participants shared similar characteristics, as they were predominantly recruited from a university population.

## Conclusion

In conclusion, based on the findings of this study, it can be inferred that a cut-off of 8 for the SPEED-vP questionnaire is the most appropriate for the Portuguese population. This cut-off point yielded a sensitivity of 71.2% and a specificity of 71%. This study further demonstrated that the SPEED-vP questionnaire is a reliable diagnostic test, showing the ability to differentiate between individuals with symptoms and those without, along with excellent internal consistency. Therefore, the SPEED-vP questionnaire can be confidently utilized in clinical settings and epidemiological research involving the Portuguese population, as it provides a valuable tool for assessing and identifying individuals experiencing symptoms related to DED.

## Data Availability

No datasets were generated or analysed during the current study.
